# Hering’s Guiding Symptoms to the Materia Medica

**Published:** 1889-02

**Authors:** 


### Hering's Guiding Symptoms to the Materia Medica.

Volumes five, six, and seven of this valuable work contain articles upon remedies from Cundurango to Natrum muriaticum, inclusive. This work is being edited by Dr. Knerr, assisted by others. Of its merits we have frequently written in the past. We cannot add more now except to say that no student of the homœopathic materia medica can afford to be without this work. It is a complementary work to Allen's *Encyclopædia;* the student needs both; he can hardly practice medicine without them. Both of these works contain errors, doubtless many of them; but, discounting all of these errors, they are invaluable to practitioners of Homœopathy.

Orders for the *Guiding Symptoms* may be sent to F. A. Davis, 1231 Filbert Street, Philadelphia.

